# Research Progress on *Salvia miltiorrhiza* Bioactive Components Regulating P‐Selectin for Microcirculatory Improvement: Potential Implications in Acute Pancreatitis

**DOI:** 10.1155/mi/4925035

**Published:** 2026-01-22

**Authors:** Yali Liu, Xinyi Ao, Weian Hao, Honglian Wang, Li Li, Shuang Wang, Jinyi Li, Jianqin Liu, Xin Zhou, Zhi Li

**Affiliations:** ^1^ Department of Spleen and Stomach Diseases, The Affiliated Traditional Chinese Medicine Hospital of Southwest Medical University, Luzhou, 646000, Sichuan, China, swmu.edu.cn; ^2^ The Key Laboratory of Integrated Traditional Chinese and Western Medicine for Prevention and Treatment of Digestive System Diseases of Luzhou City, The Affiliated Traditional Medicine Hospital of Southwest Medical University, Luzhou, 646000, China, swmu.edu.cn; ^3^ Research Center of Integrated Chinese and Western Medicine, The Affiliated Traditional Chinese Medicine Hospital of Southwest Medical University, Luzhou, Sichuan, China, swmu.edu.cn; ^4^ School of Integrated Traditional Chinese and Western Clinical Medicine, North Sichuan Medical College, Nanchong, China, nsmc.edu.cn

**Keywords:** microcirculatory dysfunction, P-selectin, *Salvia miltiorrhiza*, severe acute pancreatitis

## Abstract

Severe acute pancreatitis (SAP) is associated with high morbidity and mortality. Microcirculatory dysfunction is a critical pathological event in this process and a primary contributor to organ failure (OF). Despite the pivotal role of P‐selectin in mediating the adhesion of activated platelets and leukocytes to the vascular endothelium, a process central to microcirculatory dysfunction, effective therapeutic interventions for SAP remain limited. *Salvia miltiorrhiza*, a traditional Chinese medicinal (TCM) herb, possesses well‐documented pharmacological properties, including anti‐inflammatory, anticoagulant, and microcirculation‐improving effects. This review synthesizes recent advances in understanding the bioactive components of *Salvia miltiorrhiza*, which ameliorate microcirculation by modulating P‐selectin expression and activity through mechanisms targeting its transcription, translation, or post‐translational activation. Given the current lack of direct evidence in the context of SAP, we synthesized extensive findings from studies on cardiovascular, gastrointestinal, and inflammatory diseases, as well as from relevant acute pancreatitis (AP)/SAP models. These collective data demonstrate that *Salvia miltiorrhiza* effectively inhibits platelet aggregation, attenuates leukocyte adhesion, mitigates endothelial injury, and improves perfusion. Substantial evidence suggests that the bioactive compounds derived from *Salvia miltiorrhiza* function as effective agents against microcirculatory dysfunction by targeting P‐selectin. Leveraging this well‐defined mechanistic pathway and the promising therapeutic efficacy observed in AP/SAP models, targeting P‐selectin with *Salvia miltiorrhiza*’s bioactive compounds emerges as a compelling novel strategy for SAP‐associated microcirculatory dysfunction, laying a groundwork for subsequent validation studies.

## 1. Introduction

According to the revised Atlanta classification (2012), AP is diagnosed when at least two of the following three criteria are met: (1) characteristic abdominal pain; (2) serum amylase and/or lipase levels exceeding three times the upper limit of normal; and (3) imaging findings consistent with AP [1]. The primary etiologies of AP are gallstones and excessive alcohol consumption, each characterized by distinct pathophysiological mechanisms (Table 1) [2]. The severity of AP is categorized into three grades: mild, moderately severe, and severe acute pancreatitis (SAP). Key definitions within this classification framework include transient organ failure (OF), persistent OF, and local or systemic complications. Transient OF refers to organ dysfunction that resolves within 48 h, whereas persistent OF is defined as dysfunction lasting beyond 48 h. Local complications encompass conditions such as peripancreatic fluid collections and acute necrotic collections, while systemic complications may involve the exacerbation of pre‐existing comorbidities due to AP [1]. SAP is characterized by persistent OF, which is assessed using the modified Marshall scoring system. A score of ≥2 in any of the respiratory, cardiovascular, or renal systems that persists for more than 48 h defines this condition. Specifically, the criteria are as follows: respiratory failure is defined as a PaO_2_/FiO_2_ ratio ≤ 300; cardiovascular failure as a systolic blood pressure ≤ 90 mmHg that is unresponsive to fluid resuscitation; and renal failure as a serum creatinine level ≥ 1.9 mg/dL [1]. A global epidemiological survey on pancreatic diseases reports that the worldwide annual incidence of AP is 33.74 cases per 100,000 individuals, with an overall mortality rate of 1.16 per 100,000 population per year [6]. Among patients who develop both OF and confirmed infected pancreatic necrosis (IPN), the mortality rate can be as high as 43% [7]. Furthermore, SAP has become a critical digestive system emergency and a significant global health concern.

**Table 1:** Etiological classification of acute pancreatitis.

Etiological category	Pathophysiological mechanism	References
Gallstones (40%–70%)	Obstruction of the pancreatic duct by stones leads to enzyme activation and bile reflux	[2, 3]
Alcohol (25%–35%)	Ethanol metabolites (e.g., acetaldehyde, ethyl palmitate, and ethyl oleate) directly damage acinar cells and disrupt exocytosis	[2, 4]
Metabolic factors (~5%)	Serum triglycerides > 1000 mg/dL lead to accumulation of free fatty acids, causing acinar cell injury	[2, 5]
Others (<5%)	Includes drug‐induced toxicity or occult gallstones/microlithiasis	[2]

The pathogenesis of SAP involves a vicious cycle of aberrant pancreatic enzyme activation, inflammatory cascades, acquired coagulopathy, and microcirculatory dysfunction [8, 9]. Notably, the activation of the coagulation system is not merely a secondary event but a central driver of SAP progression. Studies have established a close association between AP severity and abnormal coagulation system activation [10–13]. Mild AP typically presents with localized pancreatic microthrombosis [14], whereas SAP can progress to disseminated intravascular coagulation (DIC), which is associated with a significantly increased mortality rate [15]. The interplay between coagulation and inflammation operates through a self‐amplifying positive feedback loop. Inflammatory cascades activate the coagulation system and suppress anticoagulant pathways; concurrently, coagulation proteases, primarily via mechanisms involving protease‐activated receptors, further amplify the inflammatory response [16, 17]. This “immunothrombosis” mechanism constitutes a critical pathological basis for microcirculatory dysfunction in SAP. Key evidence supporting this pathogenic role derives from experimental studies on anticoagulant therapy. Heparin, owing to its anti‐inflammatory properties, has demonstrated protective and therapeutic effects in both experimental models and clinical observations of AP [18, 19]. Similarly, coumarin‐based anticoagulants, such as acenocoumarol [20, 21] and warfarin [22, 23], have also demonstrated protective effects in experimental models of AP. These therapeutic findings further substantiate the pivotal role of the coagulation pathway in the pathophysiology of AP. Within this context, P‐selectin serves as a key molecular link between coagulation activation and the inflammatory cascade, playing a central role. In patients with SAP, the expression of P‐selectin on activated platelets and endothelial cells is significantly elevated, and its level correlates with disease severity. Elevated P‐selectin expression exacerbates microcirculatory perfusion impairment and tissue injury by mediating the formation of platelet‐leukocyte aggregates, enhancing leukocyte‐endothelial adhesion, and promoting the formation of neutrophil extracellular traps (NETs) [24]. In light of these mechanisms, specific laboratory biomarkers that reflect inflammatory and hemostatic balance, such as C‐reactive protein (CRP), D‐dimer, and platelet count, have demonstrated utility in predicting AP severity and mortality [25, 26].

The integrity of the microcirculation and adequate tissue perfusion are fundamental physiological prerequisites for maintaining organ function and resisting injury. Adequate blood flow ensures the delivery of oxygen and nutrients while simultaneously facilitating the clearance of locally generated toxic substances, including activated pancreatic enzymes and inflammatory mediators, thereby maintaining tissue homeostasis. This principle is well established in gastrointestinal physiology [27]. Studies demonstrate that sufficient mucosal blood flow significantly enhances the gastric mucosa’s resistance to injurious factors, whereas diminished flow markedly increases its susceptibility to damage [28]. This perfusion‐mediated protective effect appears to be a conserved mechanism across multiple digestive organs, including the oral mucosa [29], duodenum [30], colon [31], and the pancreas itself [21, 32], underscoring the universal importance of adequate blood flow in organ protection. However, during the pathogenesis of SAP, this critical protective mechanism is profoundly disrupted. A hallmark of SAP is pancreatic microcirculatory dysfunction, which manifests as capillary leakage, leukocyte stasis, and the “no‐reflow” phenomenon. These disturbances, exacerbated by ischemia‐reperfusion injury, amplify the systemic inflammatory response, thereby establishing a vicious cycle. Consequently, restoring effective organ perfusion has become a central therapeutic objective in the clinical management of SAP. Early and aggressive fluid resuscitation aims to rapidly augment the effective circulating blood volume, thereby improving organ perfusion and microcirculatory function. Current guidelines recommend an initial fluid infusion rate of 5–10 mL/kg/h, titrated until specific hemodynamic goals are achieved: a heart rate below 120 beats/min, a mean arterial pressure between 65 and 85 mmHg, and a urine output exceeding 5–10 mL/kg/h [33]. Furthermore, adequate pain management and the implementation of early enteral nutrition have been shown to confer therapeutic benefits, partly by improving splanchnic perfusion [34]. The fundamental rationale for these interventions is to counteract microcirculatory failure in SAP and to restore the intrinsic protective capacity of the affected organs.

The current management of SAP presents two major challenges: firstly, interrupting the coagulation‐inflammation vicious cycle, specifically by targeting P‐selectin as a key mediator of microcirculatory dysfunction; and secondly, identifying organ‐protective agents capable of concurrently improving blood flow perfusion and inhibiting neutrophil‐endothelial adhesion. P‐selectin, a critical molecule that links coagulation activation to the inflammatory cascade, plays a central role in the microcirculatory dysfunction associated with SAP. It is a transmembrane glycoprotein stored in platelet *α*‐granules and endothelial Weibel‐Palade bodies [35]. In the context of SAP, thrombin and inflammatory mediators activate platelets and endothelial cells, prompting the rapid translocation of P‐selectin to the cell surface. By binding to its ligand P‐selectin Glycoprotein Ligand‐1 (PSGL‐1) on neutrophils, P‐selectin mediates the formation of platelet‐leukocyte aggregates and firm leukocyte‐endothelial adhesion, thereby directly exacerbating microcirculatory perfusion impairment and tissue injury [36, 37]. Clinically, the expression of P‐selectin on activated platelets and endothelial cells is significantly elevated in SAP patients, and its level correlates with disease severity, rendering it a highly promising therapeutic target. Within this context, bioactive components derived from traditional Chinese medicine (TCM) and their modern formulations, owing to their multi‐targeted actions, demonstrate considerable therapeutic potential. Among these, *Salvia miltiorrhiza* (*Danshen*) is a representative TCM used to promote blood circulation and resolve blood stasis, and its modern pharmaceutical formulations (such as Danshen injection and sodium tanshinone IIA sulfonate injection) have been widely applied in clinical practice [38, 39]. In modern pharmacology, the traditional concept of “activating blood circulation and removing stasis” is interpreted as encompassing a spectrum of effects, including anticoagulation, inhibition of platelet aggregation, improvement of microcirculation, endothelial protection, and anti‐inflammatory activity, rather than merely augmenting cardiac output or altering peripheral vascular resistance [38]. The primary bioactive constituents of *Danshen* are categorized into water‐soluble phenolic acids, such as salvianolic acid B, and lipid‐soluble diterpenoid quinones, such as tanshinone IIA [40]. Notably, studies have demonstrated that *Danshen* can alleviate pathological injury in multiple distant organs—including the liver, lungs, kidneys, and intestines—in SAP rat models, thereby preventing the onset of multi‐OF [41–43]. This multi‐organ protective effect strongly suggests that its fundamental mechanism of action involves ameliorating systemic microcirculatory dysfunction. This review focuses on the therapeutic potential of *Danshen* and its bioactive components for SAP‐associated microcirculatory dysfunction. Although high‐quality mechanistic studies directly investigating SAP are relatively limited, substantial evidence from models of cardiovascular, cerebrovascular, and inflammatory diseases has demonstrated that *Danshen*’s bioactive components can effectively regulate the expression and function of P‐selectin through transcriptional, translational, or post‐translational mechanisms. These actions consequently inhibit platelet aggregation, reduce leukocyte‐endothelial adhesion, mitigate endothelial injury, and ultimately enhance tissue perfusion. Therefore, this review aims to integrate evidence from various disease fields to systematically elucidate the mechanisms through which *Danshen*’s bioactive components improve microcirculation by targeting P‐selectin. By inferring their substantial therapeutic potential for SAP‐associated microcirculatory dysfunction, it seeks to provide a theoretical foundation and future directions for developing novel treatment strategies.

## 2. Materials and Methods

### 2.1. Literature Source

This systematic review was conducted in strict accordance with the PRISMA (Preferred Reporting Items for Systematic Reviews and Meta‐Analyses) guidelines to govern the literature search, screening, and reporting processes. Literature searches were performed in the following databases: China National Knowledge Infrastructure (CNKI), Wanfang Data, Weipu Data, PubMed, Google Scholar, and Web of Science, spanning the period from January 1, 1989 to March 31, 2024. The objective was to comprehensively evaluate the potential of Danshen and its bioactive components to ameliorate SAP‐associated microcirculatory dysfunction through the modulation of P‐selectin.

### 2.2. Retrieval Method

A systematic and comprehensive search strategy was formulated to identify all potentially relevant literature on the topic. The search keywords were selected based on the following components: (1) Intervention: Terms related to Danshen, including its common names in Chinese and English (“danshen,” “*Salvia miltiorrhiza*”) and its frequently used formulations. (2) Core Mechanism: Terms related to P‐selectin, a key cell adhesion molecule critical for platelet activation and leukocyte recruitment, which is a potential central target for Danshen. To ensure comprehensive coverage, historical synonyms for P‐selectin, namely Granule Membrane Protein‐140 (GMP‐140) and Platelet Activation‐Dependent Granule to External Membrane Protein (PADGEM), were also included to account for their prevalence in earlier literature. (3) Target Disease: Standard terminology for SAP in both Chinese and English (“Zhongzhengjixingyixianyan,” “Severe acute pancreatitis”). (4) Target Pathological Feature: Standard terminology for microcirculatory disturbance in both Chinese and English (“Weixunhuanzhangai,” “Microcirculatory disturbance”).

The search strategy was adapted to the specific query syntax of each database. Fuzzy matching techniques were employed and supplemented by manual searching to minimize omissions. All identified records were collected and managed using Microsoft Excel 2021.

### 2.3. Literature Screening and Data Extraction

Literature screening and data extraction were performed independently by two investigators. Any disagreements were resolved through discussion with a third investigator (the corresponding author) until consensus was reached. Initially, duplicate records were removed using EndNote X9.1 software. Subsequently, the titles and abstracts of the remaining records were screened to preliminarily exclude clearly irrelevant studies. The full texts of the remaining articles were then retrieved and thoroughly assessed against the inclusion criteria.

### 2.4. Inclusion Criteria

(1) Studies involving subjects or models of SAP with microcirculatory dysfunction; (2) Studies investigating the regulatory effects of Danshen or its bioactive components on P‐selectin; (3) Experimental studies, including both in vivo and in vitro experiments; (4) Articles published in peer‐reviewed journals; (5) Original articles published in English or Chinese.

### 2.5. Exclusion Criteria

(1) Non‐primary research publications, including commentaries, editorials, conference abstracts, case reports, and review articles; (2) Studies deemed methodologically unsound, characterized by the absence of a clear control group, unjustifiably small sample size, lack of description for key experimental procedures, or the use of improper or unreported statistical methods; (3) Clinical trials or case studies involving human participants, as this review focuses on preclinical mechanistic exploration; (4) Duplicate publications or studies for which the full text was inaccessible.

### 2.6. Methodological Quality Assessment

The methodological quality and risk of bias of the included animal studies were assessed using the SYRCLE (Systematic Review Centre for Laboratory Animal Experimentation) risk of bias tool. The assessment encompassed key domains such as random sequence generation, allocation concealment, blinding of investigators and outcome assessors, random outcome assessment, incomplete outcome data, and selective reporting. The overall methodological quality of the included studies was judged to be moderate. Some studies, however, lacked detailed reporting on blinding procedures and allocation concealment.

**Figure 1:**
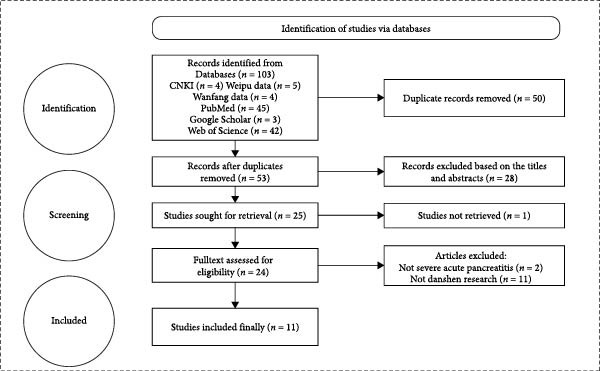
Literature search and screening process diagram.

A standardized search strategy, tailored to the specific features of each database, was developed to accurately identify relevant literature. The initial database searches retrieved 103 articles. Using EndNote X9.1, 50 duplicate records were removed. A further 28 articles were excluded after title and abstract screening, and 14 additional articles were excluded after full‐text review. Ultimately, 11 studies met all criteria and were included in the final data analysis [39, 41–50] (Table 2). The flow of the literature search and screening process is detailed in Figure 1.

**Table 2:** Summary of the basic characteristics and methodological quality assessment of the 11 included studies.

Study (author, reference number)	Study type	Model/patients	Intervention and control	Dose (standardized)	Main outcomes and assessment	Risk of bias
Ou et al. [39]	Animal experiment	Male SD rats (250–300 g); *n* = 144, 4 groups (sham, model, dexamethasone, Danshen), 12 rats/time point (3, 6, 12 h)	Intervention: Danshen injection, IV, single dose 15 min post‐modeling, continuous infusion via micro‐pump; Control: sham and model groups, equal volume saline	Dose not reported	Observation: 3, 6, 12 h. Survival: mortality lower in dexamethasone vs model; Pancreatitis severity: pathology and serum amylase; Inflammation/oxidative stress: Bax, NF‐*κ*B, TLR‐4, ICAM‐1, TUNEL staining	Moderate (randomization implemented; allocation concealment and blinding not described)

Zhang et al. [42]	Animal experiment	SD rats (250–300 g, both sexes); *n* = 192, divided into control (C), pancreatitis (P), treatment (T) groups; 4 time points: 0.5, 2, 6, 12 h (*n* = 16 per time point, 8 for lung perfusion, 8 for blood/tissue)	Intervention: 20% Danshen injection, IV (0.2 g/100 g), single dose immediately post‐modeling; Control: equal volume saline	2 g/kg	Observation: 0.5, 2, 6, 12 h. Pancreatitis severity: pathology confirmed ANP (hemorrhage, edema, necrosis), lung injury assessed; Microcirculation: lung perfusion by radioactive microspheres (^90^ᵐTc‐labeled RBCs); Hemostasis: TXA_2_/PGI_2_ ratio; Inflammation: serum PLA_2_ activity	Moderate (randomization reported; allocation concealment and blinding not described)

Zhang et al. [41]	Animal experiment	SD rats (250–300 g, both sexes); *n* = 192, same grouping as [4], 0.5, 2, 6, 12 h; *n* = 16 per time point	Intervention: 20% Danshen injection, IV (1 mL/100 g), single dose post‐modeling; Control: equal volume saline	2 g/kg	Observation: 0.5, 2, 6, 12 h. Pancreatitis severity: ANP confirmed, gastric mucosa injury assessed; Microcirculation: gastric mucosal perfusion (^99^ᵐTc‐labeled frog RBCs); Inflammation/oxidative stress: discussed but not measured	Moderate (randomization reported; allocation concealment and blinding not described)

Zhang et al. [43]	Animal experiment	SD rats (250–300 g); *n* = 96 C, P, T groups; 2, 12 h, *n* = 16 per time point	Intervention: 20% Danshen injection, IV (1 mL/100 g), single dose post‐modeling; Control: equal volume saline	2 g/kg	Observation: 2, 12 h. Pancreatitis severity: ANP confirmed, kidney injury assessed; Microcirculation: renal perfusion (^99^ᵐTc‐labeled frog RBCs); Hemostasis: TXA_2_/PGI_2_ ratio; Inflammation/oxidative stress: renal PLA_2_, neutrophil infiltration, BUN, Cr, histology	Moderate (randomization reported; allocation concealment and blinding not described)

Chen et al. [44]	Animal experiment	Male ICR (25–30 g), C57BL/6 WT and Nrf2 KO (20–25 g) mice; *n* = 8–12/group	Intervention: Tanshinone IIA, IP (5, 25, 50 mg/kg); Frog toxin model: single dose 0.5 h pre‐induction; TLC model: 2 h pre‐surgery; L‐Arg model: 24 and 48 h post first injection; Control: sham or vehicle (PBS)	5,25,50 mg/kg	Observation: frog/TLC 12 h, L‐Arg 72 h; Pancreatitis severity: HE score; Inflammation/oxidative stress: serum amylase/lipase, MDA, GSH, ROS, Nrf2/HO‐1 expression, ultrastructure (EM)	Moderate (partial randomization and blinding; allocation concealment not reported)

Li et al. [45]	Animal experiment	Female Wistar rats (250 ± 20 g); *n* = 30, 3 groups (*n* = 10)	Intervention: Danshen injection, IM (10 mL/kg) single dose post‐modeling; Control: normal and model groups, equal volume saline	10 mL/kg (original volume)	Observation: 4 h post‐surgery; Pancreatitis severity: histopathology and grading (Kyogoku for pancreas, Lei for lung/kidney); Inflammation/oxidative stress: MDA and SOD in multiple organs	Moderate (partial randomization and blinding; allocation concealment not reported)

Zhang et al. [46]	Clinical trial	67 patients with acute edematous pancreatitis (treatment 32, control 35); baseline comparable; diagnosis: ≥2 criteria (abdominal pain, elevated amylase, imaging)	Intervention: Compound Danshen injection (8–20 mL) + 5% glucose 250 mL IV once daily for 7 days; Control: conventional therapy (antispasmodic, analgesic, anti‐infective, fasting, fluid/electrolyte management)	8–20 mL/day (original volume)	Primary endpoints: clinical efficacy (cure, marked, effective, ineffective), abdominal pain relief, urine amylase recovery, hospital stay; Lab: serum P‐selectin, ICAM‐1, amylase, CBC, glucose, Ca, liver/kidney function, PaO_2_; Imaging: US for pancreatic edema	Moderate (randomization reported; allocation concealment and blinding not described)

Shi et al. [47]	Animal experiment	Male SD rats (200–250 g); AP 24, Danshen 22, sham 22	Intervention: Compound Danshen injection, IM (10 mL/kg) 30 min pre‐surgery; Control: equal volume saline	10 mL/kg (original volume)	Observation: 6 h post‐surgery; Survival: sham 4.5%, AP 25%, Danshen 13.6%; Pancreatitis severity: ascites, edema, hemorrhage/necrosis grading, HE pathology; Microcirculation: not directly measured; Inflammation/oxidative stress: plasma SOD	Moderate (randomization implemented; blinding and allocation concealment not described)

Wang et al. [48]	Animal experiment	Male SD rats (200–250 g); *n* = 50, 5 groups, *n* = 10	Intervention: Danshen injection, penile vein (4 mL/kg, diluted to 8 mL/kg), immediately and 6 h post‐surgery; Control: SAP model and sham saline, hydrocortisone group 10 mg/kg; Danshen + hydrocortisone: 4 mL/kg + 10 mg/kg	4 mL/kg (original volume)	Observation: 18 h post‐surgery; Pancreatitis severity: Kusske histopathology; Inflammation/oxidative stress: AMY, TNF‐*α*, Ca^2+^, IP_3_, intracellular Ca^2+^	Moderate (randomization implemented; allocation concealment and blinding not described)

Xiong et al. [49]	Animal experiment	Male ICR mice (8 wks, 20–23 g); Exp 1:54 mice, 3 groups (NC, SAP, Lut), *n* = 18, subdivided 6/time point; Exp 2:40 mice, 5 groups (NC, SAP, ZnPP, Lut, Lut + ZnPP), *n* = 8	Intervention: Luteolin (purity > 99%), IP 100 mg/kg, 2 h pre‐SAP induction; Control: vehicle (35% propanediol), normal saline; additional groups: ZnPP 5 mg/kg, Lut + ZnPP	100 mg/kg	Observation: 1, 3, 6 h; Pancreatitis severity: pancreas wet/dry ratio, HE score (Rongione); Inflammation/oxidative stress: serum TNF‐*α*, IL‐6, IL‐10, HO‐1; MPO, MDA, SOD; NF‐*κ*B, IkB expression	Moderate (blinding implemented; randomization and allocation concealment not fully described)

Zhang et al. [50]	Animal experiment	Male SD rats (270–330 g); SAP: 108 rats (3 × 36, 3, 6, 12 h), OJ: 180 rats (3 × 60, 7, 14, 21, 28 days)	Intervention: Danshen injection (10 mL containing equivalent of 15 g raw drug); SAP: single IV 15 min post‐surgery 0.4 mL/100 g; OJ: daily IP 0.2 mL/100 g; Control: sham and model saline	SAP: single dose of 4 mL/kg; OJ: 2 mL/kg per day.	Observation: SAP 3, 6, 12 h; OJ 7, 14, 21, 28 days; Survival: partially reported; Pancreatitis severity: gross pathology, light and electron microscopy; Microcirculation: not directly measured; Inflammation/oxidative stress: plasma endotoxin, serum TNF‐*α* (ELISA), TLR	—

Abbreviations: 6‐keto‐PGF1*α*, 6‐keto‐prostaglandin F1*α*; BUN, blood urea nitrogen; Cr, creatinine; GSH, glutathione; HO‐1, heme oxygenase 1; ICAM‐1, intercellular adhesion molecule 1; IP3, inositol trisphosphate; MDA, malondialdehyde; MPO, myeloperoxidase; NF‐*κ*B, nuclear factor kappa B; Nrf2, nuclear factor erythroid 2–related factor 2; PLA2, phospholipase A2; ROS, reactive oxygen species; SD Rat, Sprague–Dawley rat; SOD, superoxide dismutase; TLR‐4, toll‐like receptor 4; TNF‐*α*, tumor necrosis factor alpha; TUNEL, terminal deoxynucleotidyl transferase dUTP nick end labeling; TXB2, thromboxane B2.

## 3. Results

### 3.1. Regulation of P‐Selectin by Active Components of *Salvia miltiorrhiza*

The pharmacological effects of *Salvia miltiorrhiza* on the circulatory system are fundamental to the modern understanding of its traditional function of “promoting blood circulation and removing blood stasis.” Numerous in vitro and in vivo studies have demonstrated that *Salvia miltiorrhiza* and its active components can improve microcirculation and enhance blood flow through multiple mechanisms [51]. Among these mechanisms, the regulation of cell adhesion molecules, particularly P‐selectin, to inhibit leukocyte‐endothelial adhesion and platelet‐leukocyte aggregation is regarded as a key pathway through which *Salvia miltiorrhiza* alleviates microcirculatory disturbances. This action is especially relevant within the inflammatory and coagulation‐related vicious cycle of SAP. To systematically elucidate this key mechanism, we compiled direct and indirect evidence from AP/SAP‐related studies (Table 3) as well as parallel supporting evidence from other disease models (Table 4).

**Table 3:** Effects of *Salvia miltiorrhiza* and its active components on microcirculation in AP/SAP models and their potential association with the P‐selectin pathway.

Active component/formulation	Study model	Effects on P‐selectin/microcirculation‐related parameters	Potential mechanisms and association with P‐selectin pathway	References
Compound Danshen injection	Patients with acute edematous pancreatitis (clinical study)	Reduced serum P‐selectin levels	Downregulates P‐selectin expression and decreases ICAM‐1, thereby inhibiting inflammatory cell aggregation	[46]

Danshen injection (water‐soluble components)	SAP rat model (taurocholate‐induced)	Attenuated pathological damage in multiple organs (pancreas, liver, lung, kidney) and reduced mortality	Inhibits NF‐*κ*B expression and reduces apoptosis. NF‐*κ*B is a key transcriptional regulator of adhesion molecules including P‐selectin. Organ protection may partially result from NF‐*κ*B inhibition, leading to downregulation of P‐selectin and reduced inflammatory cell infiltration and microcirculatory dysfunction	[39]

Danshen injection (20% concentration)	ANP rat model (5% taurocholate‐induced)	Improved pulmonary microcirculatory perfusion (measured by radiolabeled microsphere blood flow); reduced serum PLA_2_ activity and TXA_2_/PGI_2_ ratio	Improvement in microcirculatory perfusion and modulation of TXA_2_/PGI_2_ balance represent key downstream events of P‐selectin–mediated platelet activation, aggregation, and vascular tone dysfunction. Antiplatelet and vasodilatory effects collectively contribute to amelioration of P‐selectin–related microvascular disturbances	[41]

Danshen injection (20% concentration)	ANP rat model (5% taurocholate‐induced)	Improved gastric mucosal microcirculation (increased blood flow); alleviated gastric mucosal pathological damage	Improvement in visceral (gastric mucosa) perfusion suggests attenuation of microcirculatory dysfunction caused by P‐selectin–mediated leukocyte–endothelial adhesion and vasoconstriction	[42]

Danshen injection (20% concentration)	ANP rat model (5% taurocholate‐induced)	Improved renal microcirculation (increased blood flow); reduced renal venous TXA_2_/PGI_2_ ratio, BUN, and creatinine; mitigated renal tissue damage	Improvement in renal microcirculation, modulation of local TXA_2_/PGI_2_ balance, and reduction of neutrophil infiltration reflect P‐selectin–mediated inflammatory thrombosis in the kidney, suggesting potential renal protection via this pathway	[43]

Tanshinone IIA	Three mouse AP models (caerulein, taurocholate, L‐arginine‐induced)	Reduced pancreatic and pulmonary pathological damage and serum enzyme levels	Activates Nrf2/HO‐1 antioxidant pathway, reducing ROS generation. Oxidative stress is a key upstream factor inducing endothelial and platelet P‐selectin expression, suggesting indirect inhibition of P‐selectin upregulation via potent antioxidant effects	[44]

Danshen injection	SAP rat model (taurocholate‐induced)	Attenuated multi‐organ lipid peroxidation (MDA ↓, SOD ↑)	Enhances antioxidant enzyme activity and directly scavenges free radicals. Similar to tanshinone IIA, antioxidant effects may act upstream of P‐selectin expression, indirectly modulating its levels by reducing oxidative stress	[45]

Compound Danshen injection	AP rat model (trypsin‐induced)	Reduced pancreatic hemorrhagic necrosis, mortality, and ascites volume	Demonstrates overall efficacy in AP models. Literature suggests effects may be mediated via improved pancreatic microcirculation and hypoxia tolerance, common consequences of P‐selectin dysfunction, though direct mechanistic association is not provided	[47]

Danshen injection + hydrocortisone	SAP rat model (3% taurocholate‐induced)	Reduced pancreatic intracellular IP_3_, Ca^2+^, and TNF‐*α*; mitigated pancreatic pathological damage	Modulates IP_3_/Ca^2+^ signaling to prevent calcium overload. Intracellular Ca^2+^ triggers platelet activation and P‐selectin membrane translocation, while TNF‐*α* strongly induces endothelial P‐selectin expression. Suggests dual pathway modulation of P‐selectin	[48]

Luteolin	SAP mouse model (caerulein + LPS‐induced)	Reduced pancreatic pathological damage and serum inflammatory markers; induced HO‐1 expression and inhibited NF‐*κ*B	Induces HO‐1 and inhibits NF‐*κ*B, similar to tanshinone IIA. Anti‐inflammatory and antioxidant effects may act on upstream signals regulating P‐selectin expression	[49]

Danshen injection (water‐ and lipid‐soluble components)	SAP and OJ rat models (common bile duct ligation)	Reduced plasma endotoxin and serum TNF‐*α*; improved liver function via TLR4 protein suppression	Reduces endotoxin and inhibits TLR4/TNF‐*α* pathway. Endotoxin‐TLR4 signaling is a classical upstream pathway inducing P‐selectin expression in endothelial cells and macrophages, suggesting indirect suppression of P‐selectin	[50]

**Table 4:** Supporting evidence for the regulation of P‐selectin and improvement of microcirculation by *Salvia miltiorrhiza* and its active components in other disease models.

Active component	Study model	Effect on P‐selectin	Microcirculatory improvement	Potential mechanism	References
Cryptotanshinone/Isocugenol/Tanshinone IIA	In vitro: thrombin/collagen‐activated human platelets; In vivo: FeCl_3_‐induced mouse carotid artery thrombosis model	Dose‐dependent inhibition of thrombin/collagen‐induced P‐selectin release	Inhibits platelet aggregation, adhesion, and spreading; significantly delays carotid thrombosis formation and reduces thrombus volume in vivo	Modulates Akt/ERK and cSrc/RhoA signaling pathways to inhibit platelet activation and aggregation; TSA and ISO downregulate cSrc and upregulate RhoA expression	[52]

Total phenolic acids from Danshen stems and leaves/total phenolic acids/flavonoids	Rat model of microcirculatory dysfunction induced by dextran 500	Significantly normalized abnormally elevated plasma P‐selectin levels in model rats	Improved hemorheological parameters (reduced whole blood and plasma viscosity, etc.); prolonged coagulation time; increased microvascular density in lung and thymus; regulated TXA_2_/PGI_2_ balance	Integrated anti‐inflammatory and antioxidant effects; modulates energy metabolism, oxidative stress, and inflammation‐related metabolic pathways	[53]

Salvianolic acid A	In vitro: ADP/thrombin/collagen/U46619‐activated human platelets; In vivo: photochemical injury‐induced mouse mesenteric artery thrombosis model	Inhibits ADP‐ and thrombin‐induced P‐selectin expression	Inhibits platelet adhesion on collagen and spreading on fibrinogen; significantly prolongs arterial occlusion time in wild‐type and LDLr^−^/^−^ mice	Inhibits PI3K signaling pathway, reduces Akt (Ser473/474) phosphorylation, and suppresses small GTPase Rap1 activation	[54]

Salvianolic acid B	Clinical: patients with acute coronary syndrome (ACS); In vitro: human platelets, CHO‐K1 cells expressing P2Y12 receptor	Significantly reduced ADP‐induced P‐selectin (CD62P) expression in ACS patients	Enhances standard dual antiplatelet therapy; inhibits thrombosis without affecting hemostasis	Functions as a P2Y12 receptor antagonist and phosphodiesterase (PDE) inhibitor, elevates platelet cAMP levels, enhances VASP phosphorylation, and suppresses agonist‐induced platelet aggregation and spreading	[55]

Danshen injection (mainly water‐soluble components)	Clinical: type II diabetes patients	Reduced percentage of P‐selectin‐positive platelets and improved expression	Decreased platelet activation and improved microvascular lesions	Downregulates platelet membrane P‐selectin and GP IIb–IIIa complex expression, reducing fibrinogen binding and inhibiting platelet activation	[56]

Compound Danshen dripping pills (Danshen, *Panax notoginseng*, Borneol)	Clinical: patients with unstable angina pectoris	Significant reduction in plasma GMP‐140 (P‐selectin) levels post‐treatment	Improves angina symptoms and ECG myocardial ischemia; synergistic effect with aspirin	Inhibits platelet activation; may also dilate coronary arteries, increase blood flow, and directly suppress platelet function	[57]

Compound Danshen injection (Danshen, Ligusticum chuanxiong)	Clinical: patients with traumatic cerebral infarction	Significant reduction in plasma P‐selectin levels post‐treatment	Promotes neurological recovery (improved GOS scores); improves coagulation function and microcirculation	Reduces P‐selectin, vWF, and D‐dimer levels; alleviates endothelial injury and platelet aggregation; improves hypercoagulable state	[58]

Abbreviations: cAMP, cyclic adenosine monophosphate; GOS, Glasgow Outcome Scale; GPⅡb/Ⅲa complex, integrin *α*IIb*β*3; ICAM‐1, intercellular adhesion molecule 1; VASP, vasodilator‐stimulated phosphoprotein; vWF, von Willebrand Factor.

### 3.2. Microcirculatory Improvement Mediated by P‐Selectin Inhibition

**Figure 2:**
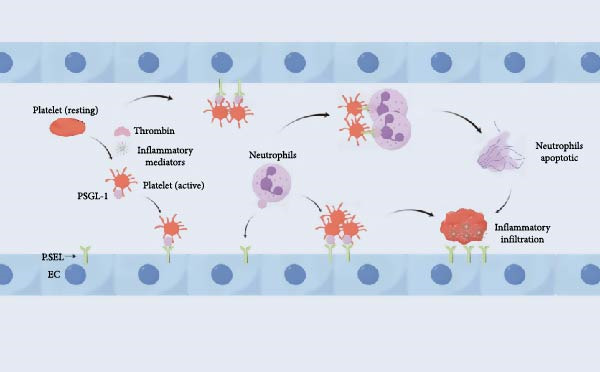
Mechanism of microcirculatory dysfunction in SAP. During SAP‐associated microcirculatory dysfunction, platelets are activated by thrombin and inflammatory mediators, leading to the rapid surface expression of P‐selectin. Its ligand, PSGL‐1, is expressed on platelets, monocytes, and neutrophils. This interaction mediates the rolling of leukocytes and platelets along the vascular wall, as well as the aggregation of platelets with neutrophils and other platelets, thereby linking inflammatory infiltration to thrombus formation. (Created with Figdraw).

SAP is characterized by OF and tissue necrosis, which primarily result from profound microcirculatory disturbances [59]. The activation of platelets and leukocytes is a key contributor to microvascular dysfunction and the infiltration of inflammatory cells into tissues [60]. The binding of P‐selectin to its high‐affinity ligand, PSGL‐1, mediates the adhesion of activated platelets and leukocytes to the vascular endothelium [61, 62]. During microcirculatory disturbances in SAP, platelets are activated by thrombin and inflammatory mediators, leading to the rapid surface expression of P‐selectin. The subsequent interaction between P‐selectin and PSGL‐1 on neutrophils induces intracellular signaling that triggers *β*2‐integrin‐dependent firm adhesion [63]. Conversely, the binding of platelet‐borne P‐selectin to neutrophil PSGL‐1 can initiate intracellular signaling within platelets, further enhancing their activation, adhesion, and the formation of microaggregates. Furthermore, endothelial P‐selectin can trigger intracellular signaling events during neutrophil adhesion, such as elevating intracellular calcium levels [64]. Clinically, these adhesive interactions among platelets, leukocytes, and endothelial cells are likely to amplify thromboinflammatory responses, thereby initiating and exacerbating microcirculatory disturbances in SAP, as illustrated in Figure 2.

### 3.3. Major Roles of *Salvia miltiorrhiza* in Improving Microcirculation in SAP

Multiple studies using experimental models of AP and SAP have demonstrated that *Salvia miltiorrhiza* and its extracts can ameliorate disease progression and confer therapeutic benefits, many of which are closely linked to improved microcirculatory function. Directly observed therapeutic effects of *Salvia miltiorrhiza* in the context of AP/SAP primarily include the mitigation of oxidative stress in the pancreas and distant organs, suppression of systemic inflammatory responses, improvement of hemorheological parameters, and attenuation of histopathological damage. Although not all studies directly quantified pancreatic microcirculation, the observed beneficial endpoint effects collectively provide robust indirect evidence supporting the efficacy of *Salvia miltiorrhiza* in improving microcirculation in SAP. Table 5 summarizes the principal bioactivities of *Salvia miltiorrhiza* validated in AP/SAP models and their corresponding observed effects.

**Table 5:** Observed effects of Danshen and its active components on microcirculation improvement in experimental AP/SAP models.

Observed effects in AP/SAP models	Extracts/compounds	Key findings in AP/SAP models	References
Reduction of oxidative stress	Danshen Extract/Injection Tanshinone IIA (TSA) Luteolin	Increased superoxide dismutase (SOD) activity in multiple organs (pancreas, liver, kidney, heart, lungs). Reduced tissue lipid peroxidation (e.g., decreased MDA levels). Decreased reactive oxygen species (ROS) generation in pancreatic tissue. Protected mitochondrial and endoplasmic reticulum ultrastructure in pancreatic acinar cells, mitigating oxidative damage. Upregulated Nrf2 and HO‐1 protein expression in pancreatic tissue.	[44, 45, 47, 49]

Alleviation of inflammatory response	Danshen Injection Compound Danshen Injection Tanshinone IIA (TSA) Luteolin	Reduced serum amylase and lipase levels. Mitigated histopathological damage in pancreas, liver, kidney, and lungs. Downregulated NF‐*κ*B protein expression in multiple organs. Decreased ICAM‐1 protein expression in lung tissue. Lowered pro‐inflammatory cytokines (TNF‐*α*, IL‐6) in serum and pancreas. Increased anti‐inflammatory cytokine IL‐10 levels. Reduced myeloperoxidase (MPO) activity in pancreatic tissue. Decreased intracellular inositol trisphosphate (IP3) and Ca^2+^ concentrations in pancreatic cells. Reduced serum phospholipase A_2_ (PLA_2_) activity. Lowered TXA_2_/PGI_2_ ratio in serum and renal venous blood. Decreased plasma endotoxin levels. Reduced serum TNF‐*α* levels. Inhibited hepatic TLR4 protein expression. Lowered serum P‐selectin levels in patients with acute edematous pancreatitis.	[39, 41, 43, 44, 46, 48–50]

Improvement of hemorheology and microvascular perfusion	Danshen Injection	Increased pulmonary tissue blood flow. Enhanced gastric mucosal blood flow. Improved renal tissue blood flow.	[41–43]

Organ protection	Danshen Injection Compound Danshen Injection Tanshinone IIA (TSA) Luteolin	Reduced mortality in experimental animals. Decreased bloody ascites and alleviated gross pancreatic hemorrhage and necrosis. Lowered apoptosis indices in multiple organs (pancreas, liver, kidney, lungs). Improved liver and kidney function indicators (reduced serum GPT, GOT, BUN, CREA levels). Mitigated SAP‐associated acute lung injury (reduced neutrophil infiltration, alveolar wall thickening, and alveolar congestion). Corrected hypocalcemia. Reduced histological damage scores in lung, gastric mucosa, and kidney tissues. Decreased neutrophil infiltration in renal tissue. Improved renal function indicators (reduced BUN and Cr levels). Ameliorated hepatic pathological damage (observed under light and electron microscopy). Shortened abdominal pain relief time, urinary amylase recovery time, and average hospital stay in patients with acute edematous pancreatitis. Improved clinical efficacy in patients with acute edematous pancreatitis (proportion of cured, markedly effective, and effective cases).	[39, 41–44, 46–50]

Abbreviations: BUN, blood urea nitrogen; Cr, creatinine; GOT, glutamic‐oxaloacetic transaminase; GPT, glutamic‐pyruvic transaminase; IL‐10, interleukin‐10; IL‐6, interleukin‐6; MPO, myeloperoxidase; NF‐*κ*B, nuclear factor kappa‐light‐chain‐enhancer of activated B cells; TLR4, toll‐like receptor 4.

## 4. Discussion

The pathogenesis and treatment of SAP have emerged as major research priorities in recent years [65]. Advances in understanding SAP pathophysiology and progress in clinical research have led to substantial improvements in its clinical management. Mortality rates for SAP have decreased substantially compared to historical data, with the majority of patients now achieving satisfactory outcomes through nonsurgical management [66]. Among the multiple pathogenic mechanisms involved, pancreatic microcirculatory disturbances are considered pivotal in initiating, sustaining, and exacerbating tissue injury, thereby playing a central role throughout the progression of SAP [53].

Current experimental and clinical evidence indicates that dysfunction of the coagulation system is a key contributor to the pathogenesis of SAP [9]. Coagulation disorders can precipitate microcirculatory failure in both the pancreas and distant organs, contributing to multi‐OF and increased mortality [24]. Upon activation by thrombin or inflammatory mediators, platelets rapidly mobilize P‐selectin to their surface. Its ligand, PSGL‐1, is expressed on platelets, monocytes, and neutrophils. This interaction mediates the rolling of leukocytes and platelets on the vascular endothelium, as well as platelet–neutrophil and platelet–platelet aggregation, thereby linking inflammatory infiltration with thrombus formation. Consequently, P‐selectin plays a pivotal role in thrombus formation by facilitating the aggregation of platelets and leukocytes on activated endothelial cells, thereby promoting the progression of acute pancreatitis.

Therefore, targeting P‐selectin represents a promising therapeutic strategy. However, the available evidence supporting this approach mainly comes from preclinical studies and is marked by substantial methodological heterogeneity. These studies differ widely in model types (e.g., caerulein‐induced, bile‐pancreatic duct ligation, ischemia–reperfusion), formulations of *Salvia miltiorrhiza* used (e.g., Danshen injection, compound preparations, tanshinone IIA), as well as in dosing regimens, routes of administration, and timing of intervention. This heterogeneity limits direct comparability of results and precludes meaningful quantitative meta‐analysis. Future studies should systematically perform subgroup analyses to compare outcomes across distinct models (e.g., edematous vs. necrotizing pancreatitis) and treatment protocols (e.g., preventive vs. therapeutic administration) to clarify the effects of *Salvia miltiorrhiza*. Furthermore, the dose–response relationships of its active components in SAP remain unclear. Elucidating these relationships would help define the optimal therapeutic window and offer more precise guidance for clinical translation.

In recent years, TCM has assumed an increasingly important role in the management of SAP. Both compound formulations and single herb preparations have demonstrated efficacy in promoting blood circulation, resolving stasis, and alleviating pain, particularly in addressing SAP‐associated microcirculatory disturbances. Studies have shown that *Salvia miltiorrhiza* can inhibit P‐selectin expression. This suppression, in turn, reduces the rolling of leukocytes and platelets in the post‐capillary venules of inflamed pancreatic microvasculature [37], prevents thrombus formation [67], and improves pancreatic microcirculation and histopathological indicators of acinar necrosis, without inducing hemorrhagic complications [35]. Therefore, *Salvia miltiorrhiza* may exert its therapeutic effects in SAP, at least in part, through the regulation of P‐selectin expression.

Studies have demonstrated that both water‐soluble phenolic acids and lipid‐soluble tanshinones from *Salvia miltiorrhiza* exhibit anti‐stasis effects in vitro and in vivo [68]. The primary antithrombotic bioactive components include, but are not limited to, salvianolic acid B, tanshinone IIA, and cryptotanshinone [38]. These components are thought to mediate their therapeutic effects in SAP primarily by improving microcirculation. The aqueous extracts of *Salvia miltiorrhiza* contain active compounds including protocatechuic aldehyde, danshensu, and rosmarinic acid [69], which confer endothelial cell protection [70] and counteract inflammation [71], calcium overload [72], and lipid peroxidation [73, 74].

In summary, while *Salvia miltiorrhiza* and its active components demonstrate promising potential for modulating P‐selectin and ameliorating microcirculatory dysfunction in SAP, several important limitations remain. First, current evidence predominantly originates from methodologically heterogeneous preclinical models—varying in induction methods, formulations, and dosing protocols—and lacks dedicated clinical trials in SAP patients. Second, the precise molecular pathways through which *Salvia miltiorrhiza* regulates P‐selectin within the complex pathophysiology of SAP are not fully elucidated. Notably, although this review focuses on mechanistic preclinical studies, clinical evidence from other conditions supports the microcirculatory benefits of *Salvia miltiorrhiza*. For instance, in acute edematous pancreatitis, compound Danshen injection significantly lowered serum P‐selectin levels, shortened the time to abdominal pain relief, and reduced hospital stay [46]. Similarly, in coronary artery disease, diabetes, and cerebrovascular disorders, Danshen preparations have been shown to improve platelet activation, endothelial function, and microcirculatory parameters [55–58]. These cross‐disease observations suggest that the effects of *Salvia miltiorrhiza* on P‐selectin and microcirculation may be generalizable, offering indirect yet valuable support for its translational potential in SAP. Moving forward, research should prioritize three directions: (1) launching prospective clinical trials—based on clarified mechanisms—to evaluate the efficacy and safety of standardized *Salvia miltiorrhiza* formulations on microcirculatory markers, P‐selectin expression, and clinical outcomes in SAP; (2) deepening mechanistic investigation using genetic tools and omics approaches to systematically map the P‐selectin‐related signaling networks modulated by Salvia miltiorrhiza in SAP; and (3) promoting standardization of preparation quality and exploring combination strategies with existing therapies. While clinical signals of microcirculatory improvement from other diseases provide a rational basis for investigating *Salvia miltiorrhiza* in SAP, this potential must be confirmed through rigorous, SAP‐specific clinical studies.

## 5. Conclusion

In summary, preclinical evidence suggests that *Salvia miltiorrhiza* and its active constituents may alleviate microcirculatory dysfunction in SAP by modulating P‐selectin expression. However, this inference is primarily based on heterogeneous animal models and in vitro studies, and its clinical translational value requires rigorous verification. A priority for future research is to elucidate the precise signaling pathways by which *Salvia miltiorrhiza* influences P‐selectin through detailed mechanistic studies and to evaluate its efficacy and safety in SAP patients via well‐designed clinical trials. Therefore, in‐depth clinical translational research, grounded in a clear understanding of the mechanism by which *Salvia miltiorrhiza* modulates P‐selectin, may offer a novel therapeutic avenue for overcoming microcirculatory disturbances in SAP.

## Disclosure

All authors read and approved the final manuscript.

## Conflicts of Interest

The authors declare no conflicts of interest.

## Author Contributions

All authors contributed to the study conception and design. **Yali Liu:** conceptualization, data curation, investigation, methodology, visualization, roles/writing – original draft, funding acquisition. **Xinyi Ao:** formal analysis, methodology. **Weian Hao:** data curation, formal analysis. **Honglian Wang:** data curation, validation. **Li Li:** data curation, supervision. **Shuang Wang:** methodology, resources. **Jinyi Li:** data curation, formal analysis. **Jianqin Liu:** software, visualization. **Xin Zhou:** funding acquisition, supervision, writing – review and editing. **Zhi Li:** funding acquisition, formal analysis, resources, software, writing – review and editing.

## Funding

This work was supported by the Science and Technology Program of Southwest Medical University (2023ZYYQ13 and 2023ZYQJ03); the Project of Sichuan Traditional Chinese Medicine Administration (2023MS416 and 2024ZD035); and the Science and Technology Program of Hospital of TCM, Southwest Medical University (2022‐CXTD‐01).

## Data Availability

All data generated or analysed during this study are included in this published article.
